# Intranasal delivery of transforming growth factor-beta1 in mice after stroke reduces infarct volume and increases neurogenesis in the subventricular zone

**DOI:** 10.1186/1471-2202-9-117

**Published:** 2008-12-10

**Authors:** Minmin Ma, Yuping Ma, Xueming Yi, Ruibing Guo, Wusheng Zhu, Xinying Fan, Gelin Xu, William H Frey, Xinfeng Liu

**Affiliations:** 1 Department of Neurology, Jinling Hospital, Nanjing University School of Medicine, 305# East Zhongshan Road, Nanjing 21002, Jiangsu Province, PR China; 2 Department of Pharmaceutics, University of Minnesota, Minneapolis, MN 55455, USA; 3 Alzheimer's Research Center, Regions Hospital, 640 Jackson Street, St. Paul, MN 55101, USA

## Abstract

**Background:**

The effect of neurotrophic factors in enhancing stroke-induced neurogenesis in the adult subventricular zone (SVZ) is limited by their poor blood-brain barrier (BBB) permeability.

Intranasal administration is a noninvasive and valid method for delivery of neuropeptides into the brain, to bypass the BBB. We investigated the effect of treatment with intranasal transforming growth factor-β1 (TGF-β1) on neurogenesis in the adult mouse SVZ following focal ischemia. The modified Neurological Severity Scores (NSS) test was used to evaluate neurological function, and infarct volumes were determined from hematoxylin-stained sections. Terminal deoxynucleotidyl transferase-mediated dUTP nick end labeling (TUNEL) labeling was performed at 7 days after middle cerebral artery occlusion (MCAO). Immunohistochemistry was used to detect bromodeoxyuridine (BrdU) and neuron- or glia-specific markers for identifying neurogenesis in the SVZ at 7, 14, 21, 28 days after MCAO.

**Results:**

Intranasal treatment of TGF-β1 shows significant improvement in neurological function and reduction of infarct volume compared with control animals. TGF-β1 treated mice had significantly less TUNEL-positive cells in the ipsilateral striatum than that in control groups. The number of BrdU-incorporated cells in the SVZ and striatum was significantly increased in the TGF-β1 treated group compared with control animals at each time point. In addition, numbers of BrdU- labeled cells coexpressed with the migrating neuroblast marker doublecortin (DCX) and the mature neuronal marker neuronal nuclei (NeuN) were significantly increased after intranasal delivery of TGF-β1, while only a few BrdU labeled cells co-stained with glial fibrillary acidic protein (GFAP).

**Conclusion:**

Intranasal administration of TGF-β1 reduces infarct volume, improves functional recovery and enhances neurogenesis in mice after stroke. Intranasal TGF-β1 may have therapeutic potential for cerebrovascular disorders.

## Background

Neurogenesis persists in the mammalian subventricular zone (SVZ) throughout adulthood. Under physiological conditions, neural stem cells from the SVZ migrate to the olfactory bulb through the rostral migratory stream (RMS) and differentiate into granule and periglomerular neurons [1]. Several studies have shown that stroke can induce neurogenesis in the adult SVZ [2,3]. Growth and neurotrophic factors, usually administered by the invasive intracerebral route, have been shown to enhance neurogenesis after stroke [4]. This suggests that they have the ability to modify endogenous neural stem cells and their potential for self-repair after ischemia.

Although neurotrophic factors contribute to adult neurogenesis, it is difficult for these large peptides (5–30 kDa) to be transported across the blood-brain barrier (BBB) into the central nervous system (CNS) [5]. Intracerebroventricular (ICV) and intraparenchymal administration are not clinically practical because of the diffusion limitations, invasiveness, cost and safety concerns [5]. Intranasal administration of growth factors provides an effective, non-invasive method for bypassing the BBB to deliver drugs to the brain along the olfactory and trigeminal neural pathways [5,6]. One recent study has demonstrated that intranasal fibroblast growth factor-2 (FGF-2) or heparin-binding epidermal growth factor-like growth factor (HB-EGF) increase neurogenesis in the normal adult mouse brain [7], but the effect of intranasal neurotrophic factors on neurogenesis in animals after stroke has not been investigated.

Transforming growth factor-β1 (TGF-β1) is a versatile cytokine capable of modulating multiple functions, such as cell growth and differentiation, inflammation and cell repair [8-10]. It also has been shown to protect neurons from various injuries, including hypoxia/ischemia, excitotoxic injury and neurotoxins [11-13]. Even though endogenous TGF-β1 expression can be up-regulated following stroke [14], ischemia-mediated elevation of TGF-β1 is insufficient and transient [15]. Whether or not TGF-β1 plays a role in the regulation of neurogenesis after brain ischemic damage remains unknown. As a peptide with large molecular weight and short plasma half-life [16], exogenous TGF-β1 cannot cross the intact BBB and may cause organ fibrosis following intravenous injection [17,18]. Intrathecal administration of TGF-β1 in mice or overexpression of TGF-β1 in the CNS in a transgenic mouse model affect the cerebrospinal fluid circulation, resulting in communicating hydrocephalus [19,20].

Our previous studies demonstrated that intranasal TGF-β1 enters the CNS where it alters gene expression [21]. Intranasal insulin-like growth factor-1 (IGF-1) has been reported to reduce infarct volume after middle cerebral artery occlusion (MCAO) and improve functional recovery [22,23]. In this study, we investigated the effects of intranasal TGF-β1 on infarct volume and neurogenesis in the adult mouse SVZ following focal cerebral ischemia.

## Results

### TGF-β1 improves neurological functional outcomes

**Figure 1:**
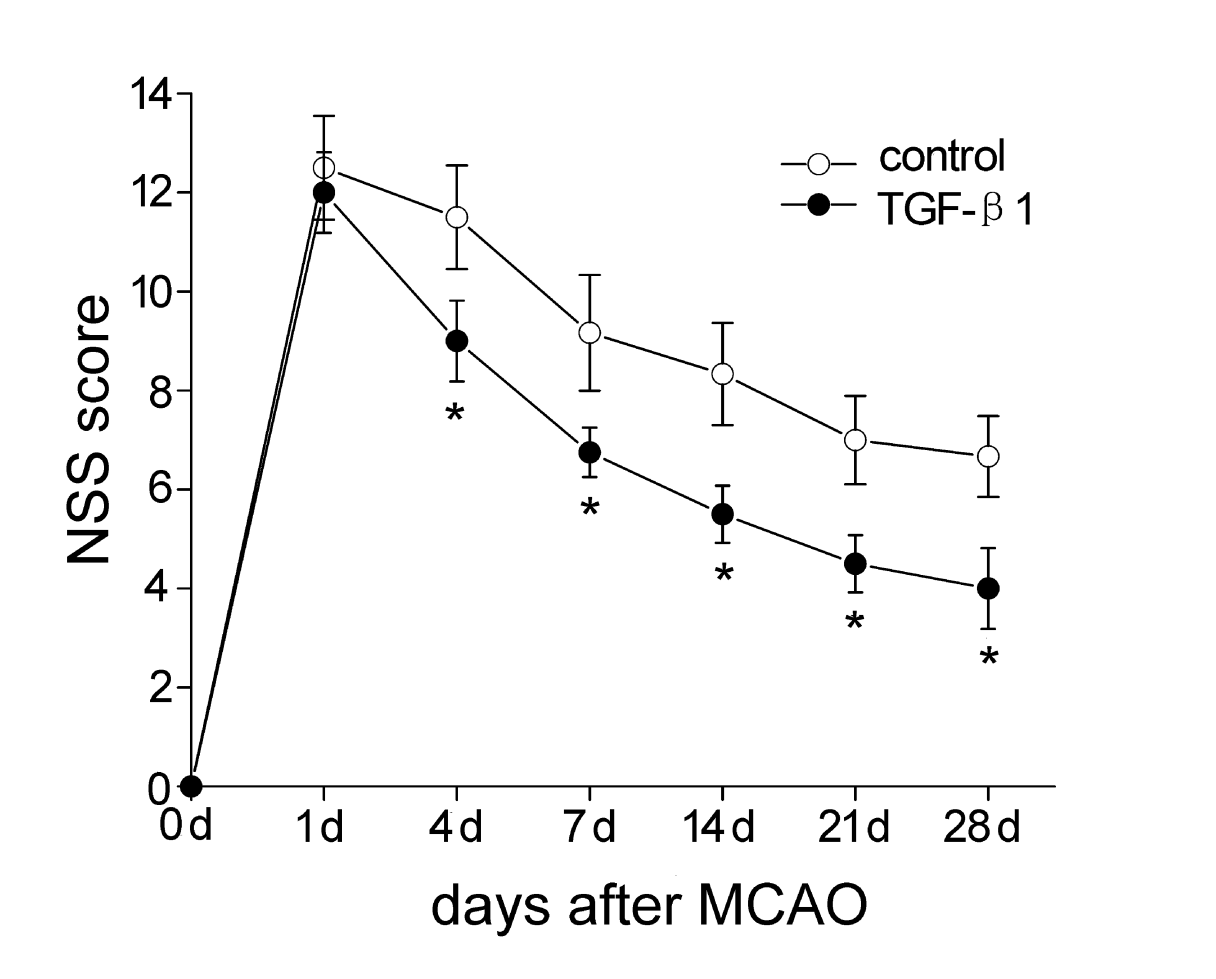
**Modified Neurological Severity Scores (NSS)**. Improved NSS were shown in mice treated with TGF-β1 (n = 4) than in control groups (n = 6) after middle cerebral artery occlusion (MCAO). Data are presented as mean ± SEM. *P < 0.05 vs. control.

To evaluate the neurological functional outcomes of TGF-β1 treatment, we applied the modified Neurological Severity Scores (NSS) test. No significant differences in NSS between control and TGF-β1 groups were observed before MCAO. All animals subjected to MCAO showed severe behavior deficits 1 day after ischemia, and there was a progressive improvement over time until 28 days after insult. However, TGF-β1-treated mice had significantly lower NSS at days 4, 7, 14, 21 and 28 after stroke, compared with the saline-treated group (*P *< 0.05) (Fig. 1).

### TGF-β1 reduces infarct volume after MCAO

**Figure 2:**
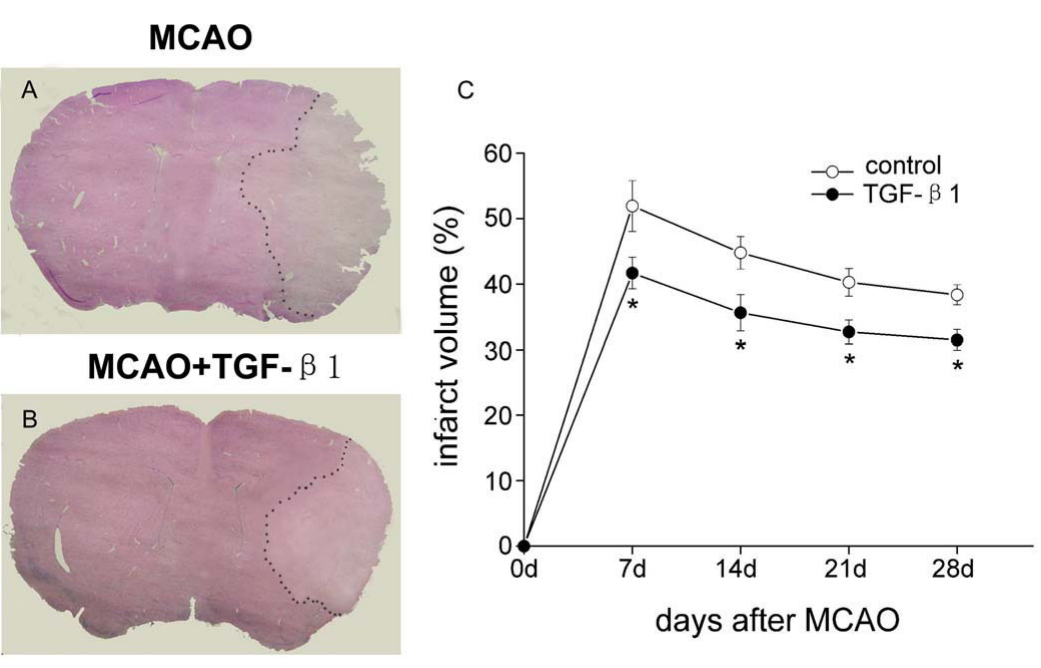
**Effects of TGF-β1 on infarct volume at 7, 14, 21 and 28 days after ischemia**. Intranasal TGF-β1 treatment group (n = 4) has reduced infarct volume (B) compared to the control group (A) (n = 6) at 7 days after MCAO. Measurement of infarct size revealed that intranasal administration of TGF-β1 significantly reduced infarct volume at days 7, 14, 21 and 28 after MCAO compared to the saline-treated groups (C). Data are presented as mean ± SEM. *P < 0.05 vs. control.

The infarct volumes measured by hematoxylin-stained sections are shown in Fig. 2.Quantitative assessment of infarct size revealed a significant reduction in infarct volume in mice receiving TGF-β1 compared to the control group at days 7, 14, 21 and 28 after MCAO (*P *< 0.05).

### TGF-β1 decreases cell death after MCAO

**Figure 3:**
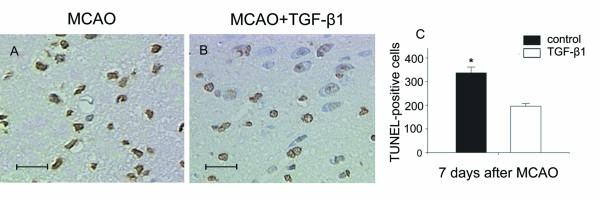
**TUNEL labeling in ipsilateral striatum at 7 days after MCAO**. TGF-β1 treated mice (n = 4) display less TUNEL-positive cells (B) than control groups (n = 6) following brain ischemia. Quantitative analysis showed reduction of the number of TUNEL-positive cells after intranasal administration of TGF-β1 (C). Scale bars = 20 μm. Data are presented as mean ± SEM. *P < 0.05 vs. control.

Terminal deoxynucleotidyl transferase-mediated dUTP nick end labeling (TUNEL) staining was preformed to confirm the antiapoptotic effect of TGF-β1. At 7 days after MCAO, a large number of TUNEL positive cells with condensed nuclei were observed in the lesioned striatum of the control groups (Fig. 3A), whereas TGF-β1 treated mice had significantly less TUNEL-positive cells in the ipsilateral striatum (Fig. 3B). Quantitative analysis showed an ~42 % of reduction of the number of TUNEL-positive cells after intranasal administration of TGF-β1 (Fig. 3C).

### TGF-β1 increases BrdU- labeled cells in SVZ and striatum

**Figure 4:**
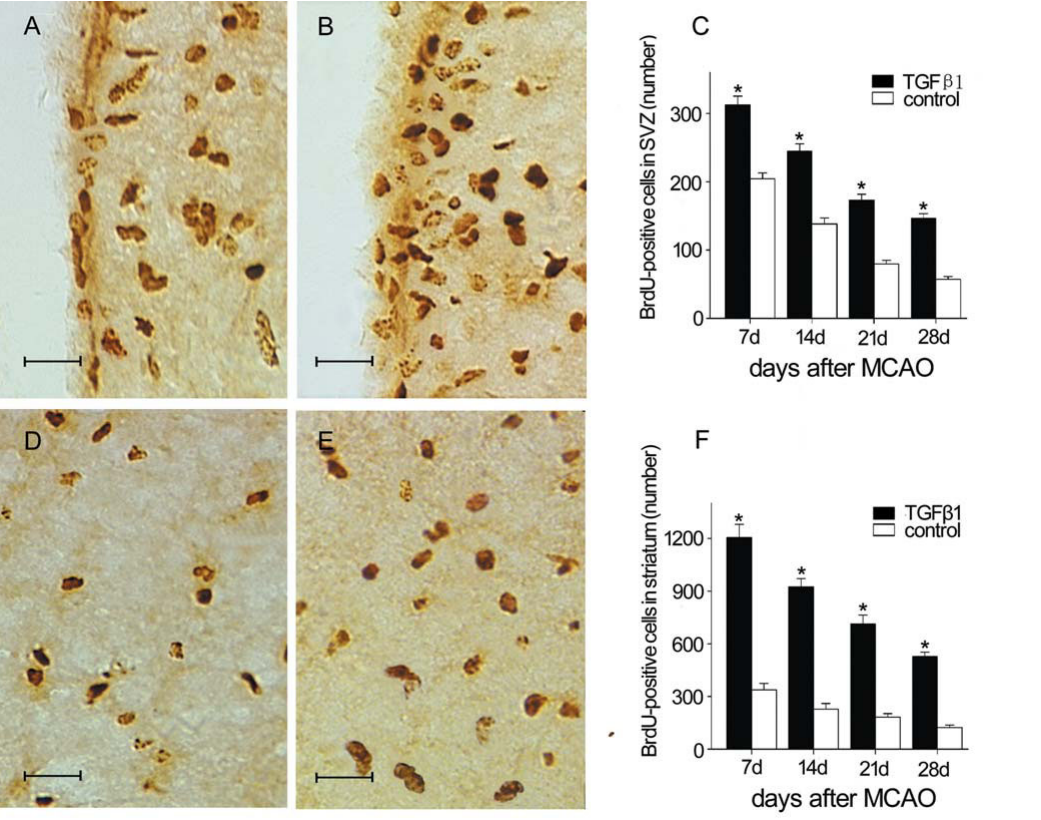
**Enhancement of BrdU incorporation by intranasal administration of TGF-β1 in adult mouse brain after MCAO**. Some BrdU-immunopositive cells were detected in the SVZ (A) and the striatum (D) of the control groups at 7 days after focal cerebral ischemia. BrdU-labeled cells were increased in the SVZ (B) and the striatum (E) by TGF-β1 intranasal treatment (n = 4) at 7 days after focal cerebral ischemia. Quantitative analysis showed that intranasal administration of TGF-β1 significantly increased the number of BrdU immunopositive cells in the SVZ (C) and the striatum (F) at 7, 14, 21 and 28 days after MCAO compared with that in the control groups (n = 6). Scale bars = 15 μm. Data are mean ± SEM. * *P *< 0.05 vs. control.

To determine whether TGF-β1 affects the amounts of progenitors in the SVZ, bromodeoxyuridine (BrdU), the thymidine analog incorporated into the DNA of dividing cells during S-phase was used to label dividing cells. Quantification of BrdU incorporated cells in the ipsilateral SVZ revealed that BrdU-labeled cells increased temporally, peaking at day 7 after stroke and declined thereafter. On day 7 post-ischemia, significantly more cells were labeled with BrdU in the SVZ of TGF-β1 -treated mice (Fig. 4B) than in that of the control mice (Fig. 4A). Significant differences were also observed between the two groups at days 14, 21 and 28 after MCAO (*P *< 0.05). Moreover, the number of BrdU labeled cells in the SVZ stabilized after day 21 with numerous BrdU-immunopositive cells present at 28 days after intranasal administration of TGF-β1 (Fig. 4C). Similar BrdU labeling was observed in the injured striatum of both groups. However, in the ipsilateral striatum at 7 days after surgery, intranasal TGF-β1 gave rise to more BrdU-positive cells (Fig. 4E) compared with saline-treated animals (Fig. 4D). Mice treated with TGF-β1 showed markedly increased numbers of BrdU-labeled cells at each time point, while the number of BrdU-positive cells remained at basal level in the control group (*P *< 0.05) (Fig. 4F).

### TGF-β1 stimulates neurogenesis in ischemic brain

To further ascertain the phenotype of BrdU-labeled cells in response to TGF-β1, we performed double immunostaining with antibodies against BrdU and other specific cell marker proteins, i.e. doublecortin (DCX), neuronal nuclei (NeuN) and glial fibrillary acidic protein (GFAP).

**Figure 5:**
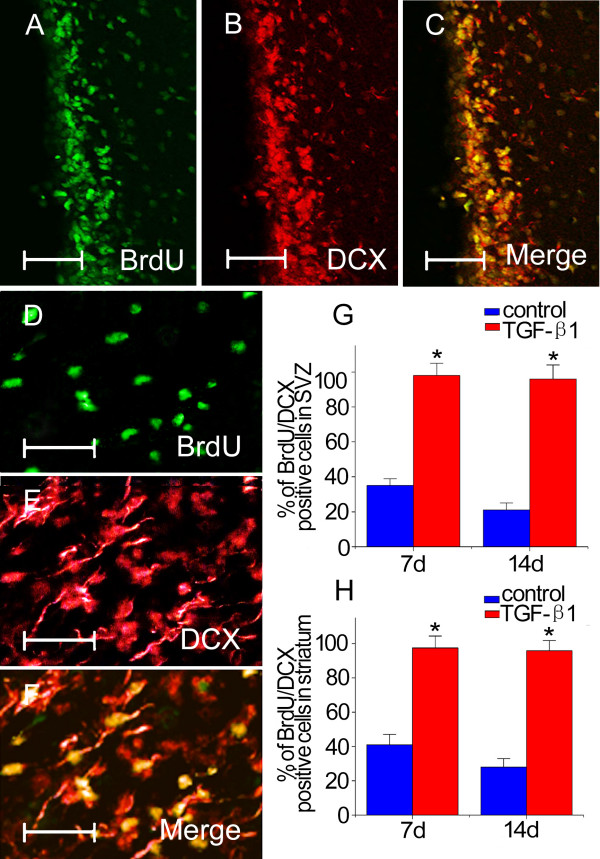
**BrdU and DCX expression in adult mouse brain**. Double immunofluorescence staining show most of BrdU-immunoreactive cells in the ipsilateral SVZ (A-C) and striatum (D-F) co-labeled with DCX after intranasal administration of TGF-β1 (n = 4) at 14 days following stroke. Quantitative data of BrdU and DCX-immunoreactive cells in the ipsilateral SVZ and striatum of TGF-β1 treated groups at 14 days after MCAO are shown in (G) and (H), respectively. Scale bars = 25 μm in A-C; 20 μm in D-F. Data are mean ± SEM. * *P *< 0.05 vs. control.

Confocal microscopy showed that BrdU immunoreactivity in ipsilateral SVZ and striatum was associated with the expression of DCX (Figs. 5A–F), but no BrdU immunopositive cell was double-labeled with NeuN or GFAP at early metaphase after ischemia in both groups. At day 7 in the control group, 35 ± 4 % and 41 ± 6 % of BrdU-labeled cells were co-labeled with DCX in the SVZ and striatum, respectively. The number of BrdU-DCX double-labeled cells declined to 21 ± 4 % and 28 ± 5 % at 14 days, respectively (Figs. 5G and 5H). In contrast to the control animals, almost complete overlap between BrdU and DCX labeling was found either at 7 days and 14 days in both regions of TGF-β1 treated mice (Figs. 5G and 5H).

**Figure 6:**
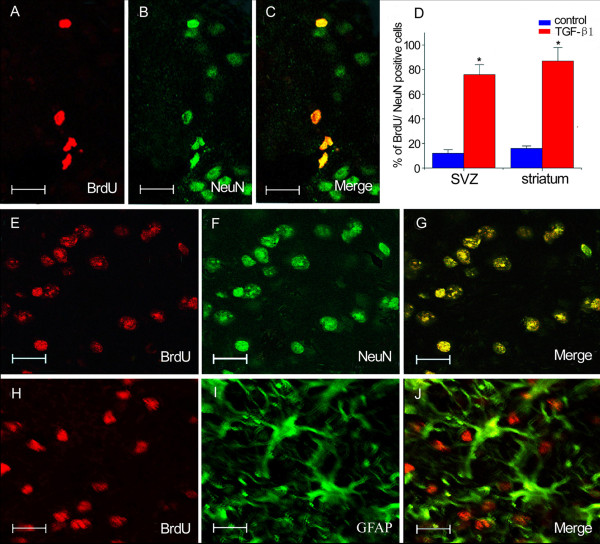
**Double immunofluorescence staining for BrdU, NeuN and GFAP**. The majority of BrdU-incorporated cells expressed NeuN in the ipsilateral SVZ (A-C) and striatum (E-G) at 28 days after stroke while only a few BrdU-positive cells colabeled with GFAP (H-J). Intranasal administration of TGF-β1 increased the number of BrdU and NeuN-immunoreactive cells in the ipsilateral SVZ and striatum (D) at 28 days after stroke. Scale bars = 15 μm in A-C; 10 μm in E-J. Data are mean ± SEM. * *P *< 0.05 vs. control.

At 28 days following the insult, 76 ± 8 % and 87 ± 11 % BrdU-incorporated cells expressed NeuN in the SVZ and striatum after intranasal administration of TGF-β1 (Figs. 6A–G), while in the mice treated with saline, only 12 ± 3 % in the SVZ and 16 ± 2 % in the striatum were co-expressed NeuN (Fig. 6D). However, there were only a few BrdU-positive cells co-labeled with GFAP in the control group (Figs. 6H–J).

## Discussion

The major finding of this study is that intranasal administration of TGF-β1 reduces infarct volume, improves functional recovery and enhances neurogenesis in the adult mouse SVZ after transient focal ischemia.

Because the delayed administration of neuroprotective agents following ischemia is usually ineffective [4], we adopted early post-ischemia intranasal treatment in this study. Our data showed that there is a significant reduction in infarct volume in mice treated with TGF-β1, which is consistent with previous findings [24]. TUNEL labeling indicated that ischemia induced DNA degradation was also decreased after intranasal administration of TGF-β1. Simultaneously, TGF-β1-treated animals exhibited progressive improvement on the NSS test compared with controls. All these support the hypothesis of a neuroprotective role for intranasal TGF-β1 in stroke. In addition to its function as a neuroprotective agent, TGF-β1 is a key regulator in development and cell cycle control [8,25], suggesting that the functional recovery after stroke may be mediated by some other mechanisms. To our knowledge, this is the first time that the effect of intranasal TGF-β1 on neuroprotection and ischemia-induced neurogenesis in the SVZ was evaluated.

Adult neurogenesis consists of one or more of the following processes, including proliferation, survival, migration and differentiation [26]. It is known that the number of BrdU immunoreactive cells in the SVZ increased with a peak at 7 days after ischemia, and then decreased gradually [27]. The loss of BrdU-positive cells may be attributed to dilution of BrdU, apoptosis, and cell migration. In the present study, we demonstrated that intranasal treatment with TGF-β1 significantly increased BrdU-labeled cells both in the SVZ and striatum ipsilateral to the ischemia at each time point from 7 days after stroke. Despite the subsequent dilution of BrdU-labeling due to continued division or death of labeled cells, we also observed an increase in BrdU-positive cells at day 21 and day 28 after MCAO. Apart from enhancing survival of newborn cells, the increased number of BrdU-labeled cells in the injured striatum of TGF-β1-treated animals may result from an accelerated migration of nascent neurons from SVZ. Different from ICV administration, TGF-β1 may localize high concentration in the striatum [21] rather than adjacent SVZ after intranasal administration, and lead to enhanced migration from SVZ into ischemic region. As expected, double immunostaining showed that after treatment with TGF-β1, most BrdU-labeled cells were co-labeled with DCX, a microtubule-associated protein expressed in migrating neuroblasts, which confirmed the previous assumption. Furthermore, we found an increased number of BrdU/NeuN double-labeled cells in the SVZ and affected striatum of TGF-β1 treated group at the anaphase after stroke, whereas only a few cells were GFAP positive, which suggest that intranasal administration of TGF-β1 also promoted progenitor's differentiation towards a neuronal lineage. Taken together, above findings indicate that stroke-induced neurogenensis is facilitated after intranasal administration of TGF-β1.

Endogenous TGF-β1 is distributed in the proliferative zone, and its two receptors TβRI, TβRII are expressed by migrating neurons and radial glia [28]. Recently, we reported that intranasal administration of TGF-β1 may exert its biological effects by regulating gene expression of TβRI and TβRII, but did not affect mRNA level of TGF-β1 itself, suggesting that the enhanced neurogenesis by TGF-β1 might be mediated through its receptors [21]. It is known that neurogenesis is linked to the cell cycle, and neural stem cells may assume their particular neuronal or glial fates by exiting the cell cycle [29]. In addition, modification of molecular morphogens and signals in the microenvironment of developing brain affects stem cell survival and differentiation [30]. TGF-β1 promotes exit from the cell cycle exit by upregulating the expression of the cell cycle protein, p21 [25]. Exogenous administration of TGF-β1 may interact with the endogenous system [31], upregulate cell adhesion molecule (CAM) expression [32], increase the potency of other neurotrophic factors involved in neurogenesis [33], modulate their action, and affect the signaling of classic neurotrophins [34], and thus enhance stroke-induced neurogenesis. The exact mechamisms for the effects of TGF-β1 on neurogenesis are still remain to be proven.

In concert with our results, a recent report showed that TGF-β1 also increased neurogenesis both in the hippocampal dentate gyrus of the adrenalectomized rats and in neural stem cells cultures even at physiological concentrations [35]. Interestingly, some data from in vivo and/or in vitro studies indicate an adverse role of TGF-β1 in regulating neurogenesis. Wachs et al. reported that TGF-β1 induced a long-term inhibition of neurogenesis in the lateral ventricular wall and the dentate gyrus after 7 days of ICV infusion to adult female Fischer-344 rats [36]. Buckwalter et al. observed that chronic overproduction of TGF-β1 also inhibited age-related neurogenesis in the hippocampus of aged transgenic mice [37]. These discrepancies may be related to the dose of TGF-β1. In contrast to other neurotrophins, TGF-β1 has been shown to produce a marked effect in a concentration-dependent manner. In developing cortex, cell migration was promoted by TGF-β1 at low concentrations whereas at high concentrations it impaired migration [31]. The decision of whether a community of progenitors undergoes predominantly neurogenesis or apoptosis is dependent on the concentration of TGF-β1 [38]. It is likely that the effect of TGF-β1 on neurogenesis is also dose-dependent, related to the concentration achieved in the brain and also to the period of time that it remains elevated.

As a non-invasive method which bypasses the BBB, intranasal administration is an alternative drug delivery strategy for targeting the brain [6]. Using this method, neuropeptides can be delivered to the brain directly from the nasal cavity for the time and treatment period needed. Two possible mechanisms shown to directly deliver drugs from the nasal mucosa to the brain along the neural pathway are the extracellular and the intracellular routes. Extracelluar transport along perineuronal and perivascular channels has been proposed to explain delivery of drugs to the brain within minutes [5,6], whereas intracellular delivery requiring internalization of the drug within the neurons followed by axonal transport takes more than a few hours for drug distribution within the brain [39]. We previously reported that TGF-β1 concentrations were significantly increased in several brain regions within 30 minutes after intranasal administration, while no increase was detected in the plasma and peripheral organs, suggesting that intranasal TGF-β1 is mainly transported into the CNS via extracellular neuronal pathways. Moreover, it was observed that the concentration of TGF-β1 following nasal delivery was sustained for at least 6 hours in some brain regions, such as striatum, thalamus, hippocampus and cortex, suggesting long-term stability of TGF-β1 tissue concentration when given via the nasal route [21]. Thus, intranasal administration of TGF-β1 represents a promising modality for facilitating neuroprotection, neurogenesis and recovery of function after stroke. Whether direct neuroprotection, neurogenesis, or both contribute to the reduction of infarct volume and the improvement in neurological function requires future investigation.

## Conclusion

Our study shows that neuroprotection, neurogenesis in the SVZ and functional recovery of the adult mouse brain are enhanced by intranasal TGF-β1 and may contribute to long-term repair following brain ischemia. Intranasal TGF-β1 may be an effective strategy for treating cerebrovascular disorders.

## Methods

### Focal cerebral ischemia procedure

Animal experiments were approved by the Animal Care Committee (Institute of Science and Technology, Jiangsu Province, China). All procedures were performed under the guideline published in the NIH *guide for the Care and Use of Laboratory Animals *(National Institutes of Health Publication No. 85-23, revised 1985). Every effort was made to minimize pain and discomfort.

Adult male C57BL/6 mice (n = 50) weighing 25–30 g (Model Animal Research Center of Nanjing University) were used for all experiments. Mice were anesthetized with chloral hydrate (10 %, 400 mg/kg, intraperitoneally). Stroke was induced by intraluminal middle cerebral artery occlusion (MCAO) with a monofilament according to Zea Longa [40]. In brief, the right common carotid artery, the right external carotid artery and the internal carotid artery were exposed via a midline incision. A 8-mm long 8-0 monofilament, with its tip rounded by heating near a flame, was advanced from the external carotid artery stump into the right internal carotid artery to occlude the origin of the right middle cerebral artery. Ninety minutes after occlusion, the filament was withdrawn to allow reperfusion. The rectal temperature was maintained at 37.0–37.5°C with a heating pad throughout the surgical procedures.

### Intranasal administration

Animals were randomly divided into control (n = 30) and TGF-β1 groups (n = 20). Recombinant human TGF-β1 (PeproTech Inc, USA) was dissolved in normal saline to a final concentration of 50 μg/ml. Intranasal administration was performed as described previously with some modifications [22,23]. Two hours after MCAO, mice were re-anesthetized with chloral hydrate (10 %, 200 mg/kg, intraperitoneally) and placed on their backs. A total volume of 20 μl solution per mouse containing 1 μg TGF-β1 or saline was given as 2 μl drops into the left and right nares, alternating sides at 2 minutes intervals over a period of 20 minutes. The mouth and the opposite naris were shut during the administration. A second series of doses was given 24 hours after initiating MCAO.

### BrdU administration

BrdU (50 mg/kg, ip; Sigma, St. Louis, MO, USA) was dissolved in saline and given intraperitoneally twice daily at 8 hour intervals for three consecutive days, starting 24 hours after initiating MCAO.

### Behavioral testing

Each mouse was subjected to the NSS test to evaluate neurological function before and at 1, 4, 7, 14, 21, 28 days after the onset of MCAO [41], and the scores were assessed by another investigator who was unaware of the experimental groups. NSS is a functional evaluation composite of motor, sensory, reflex and balance tests. The score was graded from 0 to 18 (normal score, 0; maximal deficit score, 18). Severe injury is indicated by a score of 13 to 18, moderate injury 7 to 12, and mild injury 1 to 6. In the severity scores of impairment, one point is scored for the inability to perform the task or lacking proper response for a given reflex.

### Tissue preparation and infarct volume measurement

At 7, 14, 21 or 28 days after stroke, mice (n = 6 in control group and n = 4 in TGF-β1 group at each time point) were deeply anesthetized with chloral hydrate and perfused transcardially with 150 ml saline, followed by 150 ml 4 % paraformaldehyde in 0.01 M phosphate-buffered saline (PBS) at 4°C. Brains were post-fixed in 4 % paraformaldehyde for 6 hours and cryoprotected in 30 % sucrose solution overnight. Twenty micrometer coronal sections, spaced 200 μm apart, encompassing the SVZ were cut with a cryostat and stored at -80°C.

Every 40th coronal section from total brain was stained with hematoxylin. The infarct area, and the contralateral intact hemispheric area were measured by a blinded observer using the NIH Image program. The infarct volumes were calculated by multiplying the interval thickness and presented as a percentage of the intact hemisphere, as described previously [42].

### TUNEL labeling

The brains were carefully removed at 7 days after MCAO. Five 20 μm coronal sections per brain were cut on a cryostat. TUNEL staining was preformed using a Roche assay kit (*In situ *Cell Death Detection Kit; Roche, USA), according to the manufacturer's protocol.

### Immunohistochemistry

Five to seven DAB-stained, 20 μm sections per animal were taken to evaluate the number of BrdU-labeled cells. For BrdU immunostaining, brain sections were treated with 50 % formamide, 280 mmol/L NaCl and 30 mmol/L sodium citrate at 65°C for 2 hours, incubated in 2 mol/L HCl at 37°C for 30 minutes, rinsed in 0.1 mol/L boric acid (PH 8.5) at room temperature for 10 minutes. After incubating in 3 % H_2_O_2 _for 30 minutes, sections were blocked in PBS containing 2 % goat serum, 0.3 % Triton X-100, and 0.1 % bovine serum albumin for 1 hour, followed by incubation with mouse monoclonal anti-BrdU antibody (1:800; Sigma) at 4°C overnight. Biotinylated goat anti-mouse secondary antibody (1:500; Jackson ImmunoResearch, West Grove, PA, USA) was applied for 2 hours at room temperature, then washed and incubated with peroxidase-conjugated streptavidin solution (1:500; Jackson ImmunoResearch, West Grove, PA, USA) for 30 minutes. Reaction product was detected with 3, 3'-diaminobenzidine- tetrahydrochloride (DAB, Sigma).

For double immunofluorescent staining, sections were pretreated for BrdU immunohistochemistry as described above. The following primary antibodies were used in this study: mouse monoclonal anti-BrdU antibody (1:800; Sigma), sheep polyclonal anti-BrdU antibody (1:800, Biodesign, Saco, ME), mouse monoclonal anti-NeuN antibody (1: 600, Chemicon, Temecula, CA, USA), guinea pig polyclonal anti-DCX antibody (1:3000, Chemicon), mouse monoclonal anti-GFAP antibody (1:800, Chemicon). The secondary antibodies were fluorescein isothiocyanate-conjugated goat anti-mouse IgG (1:100, Jackson ImmunoResearch), rhodamine-conjugated donkey anti-sheep IgG (1:100, Jackson ImmunoResearch), Cy3-conjugated goat anti-guinea pig IgG (1:100, Jackson ImmunoResearch). Sections were mounted with Vectashield Mounting Medium H-1000 (Vector Laboratories, Burlingame, CA, USA).

### Cell counting

Sections were viewed under high power (200×) on an Olympus BX51 microscope with Nikon digital camera, and the images were visualized on a computer monitor. The numbers of TUNEL-positive cells were counted in the impaired striatum of each animal. For BrdU-staining, only the cells with BrdU clearly localized and confined to the nucleus were considered as BrdU-reactive cells. All of the BrdU-positive cells in the lateral ventricle wall (SVZ) and striatum ipsilateral to the injury were counted. Results are presented as the average number of TUNEL or BrdU-positive cells per section.

Double-labeled cells with BrdU and a phenotype-specific marker (DCX, NeuN, or GFAP) in brain sections were identified using a confocal laser-scanning microscope (Leica TCS SP2, Germany). Only the cells in which BrdU staining was strong and clearly distributed throughout the nucleus were counted. Results are expressed as percentages of BrdU-labeled cells.

### Statistical analysis

Values for all animals of each group were averaged and standard errors of mean (SEM) were calculated for each endpoint. Statistical analysis was carried out by using two-way analysis of variance (ANOVA), followed by pairwise Student's *t*-test for modified NSS. Infarct volume and cell numbers comparisons were performed with Student's *t*-test. Probability values of < 0.05 were considered significant.

## Abbreviations

MCAO: middle cerebral artery occlusion; SVZ: subventricular zone; TGF-β1: transforming growth factor β1; BrdU: bromodeoxyuridine; NSS: Neurological Severity Scores; DCX: doublecortin; NeuN: neuronal nuclei; GFAP: glial fibrillary acidic protein; RMS: rostral migratory stream; CNS: central nervous system; BBB: blood-brain barrier; ICV: intracerebroventricular; IGF-1: insulin-like growth factor-1; FGF-2: fibroblast growth factor-2; HB-EGF: heparin-binding epidermal growth factor-like growth factor.

## Authors' contributions

XL, GX, XY and MM participated in concept and design of the study, acquisition of raw data, analysis and interpretation of data, drafting manuscript. XL provided the grant support. WF participated in technical assistance, analysis and interpretation of data and critical revision of the manuscript. YM, WZ, XF and RG carried out the animal model and the immunohistochemical study. All authors read and approved the final manuscript.

## References

[B1] Bagley J, LaRocca G, Jimenez DA, Urban NN (2007). Adult neurogenesis and specific replacement of interneuron subtypes in the mouse main olfactory bulb. BMC neuroscience.

[B2] Yamashita T, Ninomiya M, Hernandez Acosta P, Garcia-Verdugo JM, Sunabori T, Sakaguchi M, Adachi K, Kojima T, Hirota Y, Kawase T (2006). Subventricular zone-derived neuroblasts migrate and differentiate into mature neurons in the post-stroke adult striatum. J Neurosci.

[B3] Darsalia V, Heldmann U, Lindvall O, Kokaia Z (2005). Stroke-induced neurogenesis in aged brain. Stroke; a journal of cerebral circulation.

[B4] Jin K, Sun Y, Xie L, Childs J, Mao XO, Greenberg DA (2004). Post-ischemic administration of heparin-binding epidermal growth factor-like growth factor (HB-EGF) reduces infarct size and modifies neurogenesis after focal cerebral ischemia in the rat. J Cereb Blood Flow Metab.

[B5] Thorne RG, Frey WH (2001). Delivery of neurotrophic factors to the central nervous system: pharmacokinetic considerations. Clinical pharmacokinetics.

[B6] Thorne RG, Pronk GJ, Padmanabhan V, Frey WH (2004). Delivery of insulin-like growth factor-I to the rat brain and spinal cord along olfactory and trigeminal pathways following intranasal administration. Neuroscience.

[B7] Jin K, Xie L, Childs J, Sun Y, Mao XO, Logvinova A, Greenberg DA (2003). Cerebral neurogenesis is induced by intranasal administration of growth factors. Annals of neurology.

[B8] Mishra L, Derynck R, Mishra B (2005). Transforming growth factor-beta signaling in stem cells and cancer. Science.

[B9] Ishikawa M, Jin Y, Guo H, Link H, Xiao BG (1999). Nasal administration of transforming growth factor-beta1 induces dendritic cells and inhibits protracted-relapsing experimental allergic encephalomyelitis. Multiple sclerosis (Houndmills, Basingstoke, England).

[B10] Sporn MB, Roberts AB, Wakefield LM, Assoian RK (1986). Transforming growth factor-beta: biological function and chemical structure. Science.

[B11] Henrich-Noack P, Prehn JH, Krieglstein J (1996). TGF-beta 1 protects hippocampal neurons against degeneration caused by transient global ischemia. Dose-response relationship and potential neuroprotective mechanisms. Stroke; a journal of cerebral circulation.

[B12] Boche D, Cunningham C, Gauldie J, Perry VH (2003). Transforming growth factor-beta 1-mediated neuroprotection against excitotoxic injury in vivo. J Cereb Blood Flow Metab.

[B13] Krieglstein K, Suter-Crazzolara C, Fischer WH, Unsicker K (1995). TGF-beta superfamily members promote survival of midbrain dopaminergic neurons and protect them against MPP+ toxicity. The EMBO journal.

[B14] Martinez G, Di Giacomo C, Sorrenti V, Carnazza ML, Ragusa N, Barcellona ML, Vanella A (2001). Fibroblast growth factor-2 and transforming growth factor-beta1 immunostaining in rat brain after cerebral postischemic reperfusion. Journal of neuroscience research.

[B15] Zhu Y, Culmsee C, Roth-Eichhorn S, Krieglstein J (2001). Beta(2)-adrenoceptor stimulation enhances latent transforming growth factor-beta-binding protein-1 and transforming growth factor-beta1 expression in rat hippocampus after transient forebrain ischemia. Neuroscience.

[B16] Wakefield LM, Winokur TS, Hollands RS, Christopherson K, Levinson AD, Sporn MB (1990). Recombinant latent transforming growth factor beta 1 has a longer plasma half-life in rats than active transforming growth factor beta 1, and a different tissue distribution. The Journal of clinical investigation.

[B17] Kastin AJ, Akerstrom V, Pan W (2003). Circulating TGF-beta1 does not cross the intact blood-brain barrier. J Mol Neurosci.

[B18] Border WA, Noble NA (1994). Transforming growth factor beta in tissue fibrosis. The New England journal of medicine.

[B19] Tada T, Kanaji M, Kobayashi S (1994). Induction of communicating hydrocephalus in mice by intrathecal injection of human recombinant transforming growth factor-beta 1. Journal of neuroimmunology.

[B20] Zechel J, Gohil H, Lust WD, Cohen A (2002). Alterations in matrix metalloproteinase-9 levels and tissue inhibitor of matrix metalloproteinases-1 expression in a transforming growth factor-beta transgenic model of hydrocephalus. Journal of neuroscience research.

[B21] Ma YP, Ma MM, Ge S, Guo RB, Zhang HJ, Frey WH, Xu GL, Liu XF (2007). Intranasally delivered TGF-beta1 enters brain and regulates gene expressions of its receptors in rats. Brain research bulletin.

[B22] Liu XF, Fawcett JR, Thorne RG, DeFor TA, Frey WH (2001). Intranasal administration of insulin-like growth factor-I bypasses the blood-brain barrier and protects against focal cerebral ischemic damage. Journal of the neurological sciences.

[B23] Liu XF, Fawcett JR, Thorne RG, Frey WH (2001). Non-invasive intranasal insulin-like growth factor-I reduces infarct volume and improves neurologic function in rats following middle cerebral artery occlusion. Neuroscience letters.

[B24] Prehn JH, Backhauss C, Krieglstein J (1993). Transforming growth factor-beta 1 prevents glutamate neurotoxicity in rat neocortical cultures and protects mouse neocortex from ischemic injury in vivo. J Cereb Blood Flow Metab.

[B25] Siegenthaler JA, Miller MW (2005). Transforming growth factor beta 1 promotes cell cycle exit through the cyclin-dependent kinase inhibitor p21 in the developing cerebral cortex. J Neurosci.

[B26] Rola R, Mizumatsu S, Otsuka S, Morhardt DR, Noble-Haeusslein LJ, Fishman K, Potts MB, Fike JR (2006). Alterations in hippocampal neurogenesis following traumatic brain injury in mice. Experimental neurology.

[B27] Zhang RL, Zhang ZG, Zhang L, Chopp M (2001). Proliferation and differentiation of progenitor cells in the cortex and the subventricular zone in the adult rat after focal cerebral ischemia. Neuroscience.

[B28] Miller MW (2003). Expression of transforming growth factor-beta in developing rat cerebral cortex: effects of prenatal exposure to ethanol. The Journal of comparative neurology.

[B29] Ohnuma S, Harris WA (2003). Neurogenesis and the cell cycle. Neuron.

[B30] Alvarez-Buylla A, Lim DA (2004). For the long run: maintaining germinal niches in the adult brain. Neuron.

[B31] Siegenthaler JA, Miller MW (2004). Transforming growth factor beta1 modulates cell migration in rat cortex: effects of ethanol. Cereb Cortex.

[B32] Miller MW, Luo J (2002). Effects of ethanol and basic fibroblast growth factor on the transforming growth factor beta1 regulated proliferation of cortical astrocytes and C6 astrocytoma cells. Alcoholism, clinical and experimental research.

[B33] Sometani A, Kataoka H, Nitta A, Fukumitsu H, Nomoto H, Furukawa S (2001). Transforming growth factor-beta1 enhances expression of brain-derived neurotrophic factor and its receptor, TrkB, in neurons cultured from rat cerebral cortex. Journal of neuroscience research.

[B34] Unsicker K, Krieglstein K (2000). Co-activation of TGF-ss and cytokine signaling pathways are required for neurotrophic functions. Cytokine & growth factor reviews.

[B35] Battista D, Ferrari CC, Gage FH, Pitossi FJ (2006). Neurogenic niche modulation by activated microglia: transforming growth factor beta increases neurogenesis in the adult dentate gyrus. The European journal of neuroscience.

[B36] Wachs FP, Winner B, Couillard-Despres S, Schiller T, Aigner R, Winkler J, Bogdahn U, Aigner L (2006). Transforming growth factor-beta1 is a negative modulator of adult neurogenesis. J Neuropathol Exp Neurol.

[B37] Buckwalter MS, Yamane M, Coleman BS, Ormerod BK, Chin JT, Palmer T, Wyss-Coray T (2006). Chronically increased transforming growth factor-beta1 strongly inhibits hippocampal neurogenesis in aged mice. The American journal of pathology.

[B38] Hagedorn L, Floris J, Suter U, Sommer L (2000). Autonomic neurogenesis and apoptosis are alternative fates of progenitor cell communities induced by TGFbeta. Developmental biology.

[B39] Shipley MT (1985). Transport of molecules from nose to brain: transneuronal anterograde and retrograde labeling in the rat olfactory system by wheat germ agglutinin-horseradish peroxidase applied to the nasal epithelium. Brain research bulletin.

[B40] Longa EZ, Weinstein PR, Carlson S, Cummins R (1989). Reversible middle cerebral artery occlusion without craniectomy in rats. Stroke; a journal of cerebral circulation.

[B41] Chen J, Li Y, Wang L, Zhang Z, Lu D, Lu M, Chopp M (2001). Therapeutic benefit of intravenous administration of bone marrow stromal cells after cerebral ischemia in rats. Stroke; a journal of cerebral circulation.

[B42] Swanson RA, Morton MT, Tsao-Wu G, Savalos RA, Davidson C, Sharp FR (1990). A semiautomated method for measuring brain infarct volume. J Cereb Blood Flow Metab.

